# New Biocalcifying Marine Bacterial Strains Isolated from Calcareous Deposits and Immediate Surroundings

**DOI:** 10.3390/microorganisms10010076

**Published:** 2021-12-30

**Authors:** Julia Vincent, Béatrice Colin, Isabelle Lanneluc, René Sabot, Valérie Sopéna, Philippe Turcry, Pierre-Yves Mahieux, Philippe Refait, Marc Jeannin, Sophie Sablé

**Affiliations:** 1 Laboratoire Littoral Environnement et Sociétés, La Rochelle Université, UMR 7266 CNRS, 17000 La Rochelle, France; julia.vincent1@univ-lr.fr (J.V.); beatrice.colin@univ-lr.fr (B.C.); isabelle.lanneluc@univ-lr.fr (I.L.); valerie.gauthier@univ-lr.fr (V.S.); 2 Laboratoire des Sciences de l’Ingénieur pour l’Environnement, La Rochelle Université, UMR 7356 CNRS, 17000 La Rochelle, France; rene.sabot@univ-lr.fr (R.S.); philippe.turcry@univ-lr.fr (P.T.); pierre-yves.mahieux@univ-lr.fr (P.-Y.M.); philippe.refait@univ-lr.fr (P.R.)

**Keywords:** biocalcifying marine bacteria, calcite, urease, carbonic anhydrase, *Virgibacillus*, *Pseudoalteromonas*, *Pseudidiomarina*, *Epibacterium*, *Planococcus*, *Bhargavaea*

## Abstract

Marine bacterial biomineralisation by CaCO_3_ precipitation provides natural limestone structures, like beachrocks and stromatolites. Calcareous deposits can also be abiotically formed in seawater at the surface of steel grids under cathodic polarisation. In this work, we showed that this mineral-rich alkaline environment harbours bacteria belonging to different genera able to induce CaCO_3_ precipitation. We previously isolated 14 biocalcifying marine bacteria from electrochemically formed calcareous deposits and their immediate environment. By microscopy and µ-Raman spectroscopy, these bacterial strains were shown to produce calcite-type CaCO_3_. Identification by 16S rDNA sequencing provided between 98.5 and 100% identity with genera *Pseudoalteromonas, Pseudidiomarina, Epibacterium, Virgibacillus, Planococcus,* and *Bhargavaea*. All 14 strains produced carbonic anhydrase, and six were urease positive. Both proteins are major enzymes involved in the biocalcification process. However, this does not preclude that one or more other metabolisms could also be involved in the process. In the presence of urea, *Virgibacillus halodenitrificans* CD6 exhibited the most efficient precipitation of CaCO_3_. However, the urease pathway has the disadvantage of producing ammonia, a toxic molecule. We showed herein that different marine bacteria could induce CaCO_3_ precipitation without urea. These bacteria could then be used for eco-friendly applications, e.g., the formation of bio-cements to strengthen dikes and delay coastal erosion.

## 1. Introduction

Natural limestone structures formed under the action of microorganisms, such as beachrocks and stromatolites, are observed in the marine environment [1,2]. Indeed, microbial activity can provide an optimal microenvironment for mineral precipitation [3]. This phenomenon, called biomineralisation, can lead to the formation of biominerals mainly composed of carbonates, phosphates, sulphates, and silicates, combined with bivalent cations, such as calcium, magnesium, iron, and/or manganese [4]. Most of these biominerals are calcium compounds, among which calcium carbonate phases (CaCO_3_) are the most abundant [3]. CaCO_3_ precipitation induced by microorganisms (biocalcification) plays a significant role in natural cementing processes in caves, soils, and sediments, both in marine and freshwater environments [3]. The biocalcification process involves a wide range of bacterial species with different metabolisms [5,6,7]. The main microbial metabolic pathways involved in CaCO_3_ precipitation are urea hydrolysis, ammonification of amino acids, denitrification, photosynthesis, methane oxidation, sulphate reduction, and oxidation of organic compounds [3,5,6,7]. All these metabolic pathways have common factors: production of CO_2_ or carbonates, release or presence of calcium ions in the medium, and an alkalinisation that favours CaCO_3_ precipitation. Among these metabolic pathways, urea hydrolysis is the best known and the most efficient biocalcification process for CaCO_3_ [8,9,10,11,12,13,14]. The efficiency of urease activity has been demonstrated in bacteria, such as *Sporosarcina pasteurii* and *Bacillus sphaericus* [15,16,17,18]. During microbial urease activity, the hydrolysis of urea is catalysed by urease enzymes and produces carbamate and ammonia (Equation (1)). Further, from the spontaneous hydrolysis of carbamate, carbonic acid and additional ammonia are produced (Equation (2)). Ammonia then reacts with water and produces ammonium and hydroxide ions (Equation (3)). Consequently, pH is increased around the cell and induces precipitation of calcium carbonate if soluble Ca(II) species are present (Equations (4) and (5)) [6].

CO(NH_2_)_2_ + H_2_O → NH_2_COOH + NH_3_ (1)

NH_2_COOH + H_2_O → NH_3_ + H_2_CO_3_ (2)

2NH_3_ + 2H_2_O → 2NH_4_^+^ + 2OH^−^ (3)

2OH^−^ + H_2_CO_3_ → CO_3_^2−^ + 2H_2_O (4)

Ca^2+^ + CO_3_^2−^ → CaCO_3_ (5)

Another metabolic pathway for biocalcification involves the carbonic anhydrase (CA) enzyme [19,20]. This widespread enzyme is involved in several physiological processes related to CO_2_ and carbonates, such as respiration, CO_2_ transport, and photosynthesis [21]. Nevertheless, few studies have been conducted on prokaryotes concerning the direct involvement of this enzyme in CaCO_3_ precipitation [11,22]. CA promotes the interconversion of CO_2_ into HCO_3_^−^ (Equation (6)) and increases pH by releasing carbonates and bicarbonates in large quantities [3,4].

H_2_O + CO_2_ ↔ HCO_3_^−^ + H^+^ (6)

Under alkaline conditions and in the presence of dissolved calcium, bicarbonates formed by CA can precipitate as CaCO_3_ [4,23,24]. Therefore, CA and urease can act synergistically [11,19]. Owing to an increase in pH due to urease activity, a pool of carbonate ions provided by CA and the presence of dissolved calcium, production of CaCO_3_ can be enhanced.

CaCO_3_ precipitation also occurs in abiotic conditions on metal surfaces under cathodic polarisation in seawater. A calcareous deposit made of a mixture of CaCO_3_ and magnesium hydroxide Mg(OH)_2_ is formed because the cathodic reactions taking place at the metal surface, i.e., O_2_ reduction and/or H_2_O reduction, produce OH^−^ ions and increase the pH at the metal/seawater interface [25]. In the marine environment, such calcareous deposits act as a cement between sediments, shells, and pebbles and give rise to a solid agglomerate that could reinforce dikes and delay the erosion of beaches [26,27]. In a previous preliminary work [28], we showed for the first time that this CaCO_3_-rich environment could host microorganisms and that it should be explored to isolate indigenous bacteria that can survive in extreme alkaline environments for efficient biocalcification. Fourteen marine bacteria able to induce CaCO_3_ precipitation were thus isolated from calcareous deposits developed by cathodic polarisation on steel grids and their immediate environment (mud and seawater).

In the present work, we carried out a thorough characterisation of these 14 strains while specifying the characteristics of the environment from which they were isolated. All the strains were identified based on molecular characteristics. Investigations were conducted to validate urease and/or CA activities and their role in biocalcification. Finally, the biocalcification capacity of each of the isolated marine bacteria was evaluated by monitoring bacterial growth, pH evolution, and CaCO_3_ production for 7 days.

## 2. Materials and Methods

### 2.1. Bacterial Strains and Growth Conditions

The biocalcifying marine bacterial strains were grown at 30 °C in marine broth (MB) medium (Condalab, Madrid, Spain) containing ammonium nitrate 0.0016 g L^−^^1^, bacteriological peptone 5 g L^−1^, boric acid 0.022 g L^−1^, anhydrous calcium chloride 1.8 g L^−1^, disodium phosphate 0.008 g L^−1^, anhydrous magnesium chloride 8.8 g L^−1^, potassium bromide 0.08 g L^−1^, potassium chloride 0.55 g L^−1^, sodium bicarbonate 0.16 g L^−1^, sodium chloride 19.4 g L^−1^, sodium fluoride 0.0024 g L^−1^, sodium silicate 0.004 g L^−1^, sodium sulphate 3.24 g L^−1^, strontium chloride 0.034 g L^−1^, yeast extract 1 g L^−1^, and ferric citrate 0.1 g L^−1^, pH 7.6 ± 0.2, or in M1 medium (adapted from Silva-Castro et al., 2015 [29]) containing yeast extract 10 g L^−1^, peptone 5 g L^−1^, glucose 1 g L^−1^, and sea salts 30 g L^−1^, pH 7.2.

*Alcanivorax borkumensis* DSM 11573 [30], a marine bacterial strain selected for its ability to biocalcify, was used as a control and grown at 30 °C in M809 medium containing sodium chloride 23 g L^−1^, magnesium sulphate heptahydrate 5.8 g L^−1^, magnesium chloride dihydrate 6.16 g L^−1^, calcium chloride dihydrate 1.47 g L^−1^, sodium hydrogen phosphate heptahydrate 0.89 g L^−1^, sodium nitrate 5 g L^−1^, ferrous sulphate heptahydrate 0.03 g L^−1^, and sodium pyruvate 10 g L^−1^, pH 7.0–7.5. 

*Sporosarcina pasteurii* DSM 33, a non-marine bacterial strain, also used as a control for its ability to biocalcify [8,9], was grown at 30 °C in M220 + 2% urea composed of peptone from casein 15 g L^−1^, peptone from soymeal 5 g L^−1^, sodium chloride 5 g L^−1^, and urea 20 g L^−1^, pH 7.3.

The non-marine bacterial strain *Escherichia coli* ATCC 25922, used as a control for its urease-negative and carbonic anhydrase-positive characteristics, was grown at 37 °C in Tryptic soy Broth medium (TSB) composed of tryptone 17 g L^−1^, papain digest of soybean meal 3 g L^−1^, glucose 2.5 g L^−1^, dipotassium phosphate 2.5 g L^−1^, and sodium chloride 5 g L^−1^, pH 7.3 ± 0.2. 

The different media were supplemented or not with calcium chloride dihydrate 3.7 g L^−1^ (20 mmol L^−1^) and/or urea 20 g L^−1^ (330 mmol L^−1^). Solid media were prepared by adding agar 12 g L^−1^. For liquid cultures, all bacterial strains were grown with shaking (130 rpm).

Artificial seawater (ASW) based on the standard ASTM D1141 (standard specification for substitute ocean water, corrosion testing procedures, 1992) was composed of sodium chloride 24.54 g L^−1^, calcium chloride dihydrate 1.54 g L^−1^, magnesium chloride hexahydrate 11.1 g L^−1^, sodium sulphate 4.09 g L^−1^, potassium chloride 0.69 g L^−1^, and sodium carbonate 0.23 g L^−1^.

### 2.2. Sampling and Isolation of Biocalcifying Bacterial Strains 

Biocalcification-inducing marine bacterial strains were previously collected from natural seawater (SW), mud (MD), and calcareous deposits (CD) in the Atlantic Ocean from two different sites in the La Rochelle area (France, La Rochelle Harbour, 46.14299° N, 1.16544° W and Angoulins, 46.10267° N, 1.12222° W) [28]. The same protocol was used for the samples collected from each site. The calcareous deposit considered for this study was previously developed on steel grids by cathodic polarisation applied for six months at a constant current density of −150 µA cm^−2^. Samples of about 10 mL of seawater, 10 g of mud and 8 g of calcareous deposit were collected in November 2018. Seawater was collected from above the polarised grid in a sterile bottle. Polarised grids were deposited in a container of seawater from their environment, transported to the laboratory, and immediately processed. The mud was then collected from the grids using a sterile scalpel and transferred to a sterile vial. The calcareous deposit was sterilely scraped from different points of the grids with a scalpel, homogenised by grinding, and transferred into a sterile bottle. A first half of each sample was then placed in 50 mL of MB, and the second half was placed in 50 mL of M1 medium, both media being supplemented with CaCl_2_ 20 mmol L^−1^. All samples were thereafter incubated at 25 °C with shaking (130 rpm, shaker Ecotron Infors HT, Massy, France) for one week. For each culture condition, bacterial cultures were diluted in ASW using the serial dilution technique, and each dilution was plated on solid-media MB and M1, supplemented with CaCl_2_ 20 mmol L^−1^, and incubated for 3 days at 25 °C. By observation with a binocular magnifying glass (see Section 2.6.1), bacterial colonies showing mineral precipitation on their surface were selected. These bacteria were purified by successive subculturing and stored in 25% glycerol at −80 °C before analysis. As no significant difference in growth was observed between MB and M1 media, all selected strains were subsequently grown at 30 °C in MB.

### 2.3. DNA Extraction, 16S rRNA Gene Amplification (PCR) and Sequencing, and Bacterial Strain Identification

Genomic DNA was extracted from pure culture using the Genomic DNA from Tissue Kit (Macherey Nagel, Hoerdt, France) according to the instructions of the manufacturer. Amplification of about 1400 bp 16S rRNA gene was performed in 50 µL of PCR reaction mixture containing 50–100 ng template DNA, 0.2 µmol L^−1^ of each primer (16SUnivF, _5′_AGAGTTTGATCCTGGCTCA_3′_ and 16SUnivR, _5′_GGCTACCTTGTTACGACTT_3′_ [31], 320 µmol L^−1^ dNTP, 3 mmol L^−1^ MgCl_2_, and 0.04 U Taq DNA polymerase in the corresponding 1× buffer (Fermentas, Waltham, MA, USA). Polymerase chain reaction was performed in a thermal cycler under the following conditions: 95 °C for 2 min, 30 cycles of 30 s at 92 °C, 30 s at 54 °C, and 1.5 min at 72 °C. Amplicons were sent to GenoScreen (Lille, France) for purification and gene sequencing. Amplicons with insufficient sequence quality were remade by Genoscreen with their own primers (P8/P535 and/or 1040F/1040R) and resequenced. 

To identify the isolated bacterial strains, the 16S rDNA sequences were compared with those in GenBank using Blast software (http://blast.ncbi.nlm.nih.gov/Blast.cgi (accessed on 10 March 2021)). Genus identification of these bacteria was confirmed using SILVA ACT (https://www.arb-silva.de/aligner/ (accessed on 15 December 2021)) and RDP II classifier (http://rdp.cme.msu.edu/classifier/classifier.jsp (accessed on 15 December 2021)). Sequences were then submitted to the GenBank database (accession numbers MW774399 to MW774412).

### 2.4. Microbial Growth Kinetics and Calcium Carbonate Production

#### 2.4.1. Culture Conditions

For each bacterial strain, growth kinetics were carried out during 7 days of incubation at 30 °C with shaking (130 rpm, shaker Ecotron Infors HT) in three different culture media: MB, MB supplemented with 20 mmol L^−1^ CaCl_2_ (MB + CaCl_2_), and MB + CaCl_2_ supplemented with 330 mmol L^−1^ urea (MB + CaCl_2_ + urea). For each condition, 100 mL of culture media was inoculated with bacteria at 1% from an overnight culture in MB, and 500 µL was collected for analysis regularly over 7 days of incubation. The pH of each sample was measured using a WTW pH-meter 3310 with electrode SENTIX Mic-D (WTW). Bacterial enumeration was carried out by the method of serial dilution of the samples. The samples were diluted in ASW and plated on solid MB medium for 24 h at 30 °C.

#### 2.4.2. Collection of Calcium Carbonate Precipitates

Bacterial strains were grown under the same three conditions as for growth kinetics (see Section 2.4.1). After 7 days of incubation, the whole cultures (100 mL) were centrifuged at 2500× *g* for 5 min. The pellets, which included bacterial cells and calcium carbonate precipitates, were washed in 20 mL of sterile water, centrifuged again, and resuspended in 20 mL of sterile water. Samples were then frozen at −80 °C and lyophilsed by a free-drying process at a pressure below 450 mTorr at −80 °C (COSMOS 20K) for 4 days. 

#### 2.4.3. Thermogravimetric Analysis

Quantification of bacterial CaCO_3_ production was performed by thermogravimetric analysis (TGA) on a Setaram Setsys Evolution 16/18 apparatus. Quantities of 20 to 60 mg of dried samples were heated from 20 °C to 1000 °C at a 10 °C/min heating rate under a neutral argon atmosphere. CaCO_3_ content was deduced from the mass loss between 550 and 900 °C due to decarbonation. To obtain a more accurate temperature range, data were analysed through differential thermogravimetric curves.

### 2.5. Morphological and Metabolic Characterisation of Bacteria

#### 2.5.1. Morphological Characterisation

The morphology of colonies and cells was characterised. Gram staining was performed according to the standard method [28]. 

#### 2.5.2. Urease Activity Assay

Urease activity assay was performed using bacterial strains from an overnight culture in MB at 30 °C and indole urea medium (Biomérieux, Craponne, France) according to the instructions of the manufacturer. Two tests were performed at room temperature: (i) In the first assay, 100 µL of bacterial cultures was inoculated in 900 µL of indole urea medium; (ii) In the second assay, after a cell concentration step by centrifugation of the bacterial cultures at 2500× *g* for 5 min, the cell pellets were resuspended in 1 mL of fresh indole urea medium. The presence of urease activity was determined by observation of the colour change (yellow to pink) induced by the increase in pH following the release of ammonium from urea. *Escherichia coli* ATCC 25,922 and *Sporosarcina pasteurii* DSM 33 were used as negative and positive controls of urease activity, respectively.

#### 2.5.3. Carbonic Anhydrase Activity Assay

Esterase activity of carbonic anhydrase (CA) was detected using *p*-nitrophenyl acetate (*p*-NPA, Sigma-Aldrich, Saint-Quentin-Fallavier, France) as CA substrate [32]. The reaction solution was composed of 0.3 mmol L^−1^ of *p*-NPA and 20 mmol L^−1^ of sodium phosphate buffer (pH 7.5). Bacterial colonies grown on solid MB for 24 h were added to the reaction solution (1 mL) and incubated for one hour at room temperature. The esterase activity of CA was detected by a colour change of the solution, which turns yellow when *p*-NPA is hydrolysed. For each experiment, the reaction solution without bacteria was used as a negative control, and *Escherichia coli* ATCC 25955 was used as a positive control of CA activity.

#### 2.5.4. Inhibition of Carbonic Anhydrase Activity 

The protocol was adapted from Krause et al. [30]. Two sulphonamides, acetazolamide (AZ), and ethoxzolamide (EZ) (Sigma-Aldrich) were used for CA inhibition. Stock solutions of AZ (625 mmol L^−1^) and EZ (1250 mmol L^−1^) were prepared in N,N-dimethylformamide (DMF). For inhibition experiments, solid MB medium in Petri dishes containing AZ or EZ at a final concentration of 100 µmol L^−1^ was inoculated on the surface with 10 µL of an overnight culture in MB. After 48 h of incubation at 30 °C, crystal enumeration on the surface of bacterial colonies was carried out using fluorescence microscopy (see Section 2.6.2). *Alcanivorax borkumensis* DSM 11573 was used as a positive control [30].

### 2.6. Crystal Morphology and Identification

#### 2.6.1. Binocular Magnifier

Morphology of mineralisation products at the surface of the bacterial colonies grown on solid MB at 30 °C for at least 3 days were observed with a binocular magnifying glass Leica M165 C (12.5× to 120×).

#### 2.6.2. Fluorescence Microscopy

Mineralisation products were observed using fluorescence microscope (ZEISS Axio Observer, ZEISS, Marly-Le-Roi, France) with magnification of 50× and 200× in brightfield and under fluorescence (UV-A filter, emission 350 nm; excitation 470 nm). ZEN software (ZEISS) was used for image acquisition, and images were analysed using Fiji/Image J software.

#### 2.6.3. Raman Spectroscopy

μ-Raman spectroscopy analysis was used for crystal identification and performed at room temperature on a Jobin Yvon high-resolution Raman spectrometer (LabRAM HR Evolution, HORIBA, Longjumeau, France) equipped with a microscope (Olympus BX 41, Olympus, Rungis, France), a Peltier-based cooled, charge-coupled detector (CCD), and a near-infrared diode laser (785 nm). The acquisition time was variable and depended on fluorescence background of the spectrum due to the organic matter. It was generally equal to 60 s but could be increased up to 5 min to optimise the signal-to-noise ratio. At least 5 or 6 zones (diameter of approximately 2 μm) of a given deposit were analysed through a long-working-distance 50× objective, with a spectral resolution estimated at 0.5 cm^−1^.

### 2.7. Statistical Analyses 

All values presented in the Results and Discussion section are the averages of data from three independent experiments. Statistical analyses were performed using GraphPad Prism version 5.00 software using two-way ANOVA analysis and Bonferroni post hoc tests. Differences between a sample and the corresponding control were considered significant if *p* values were <0.05.

## 3. Results and Discussion

### 3.1. Biocalcifying Marine Bacteria

**Figure 1:**
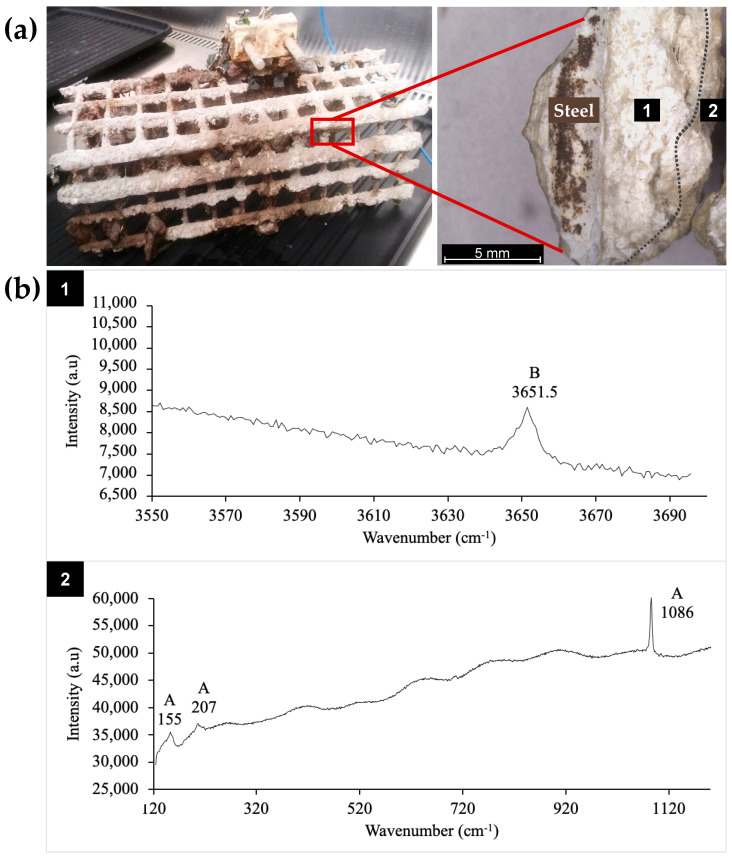
(**a**) Calcareous deposit obtained by cathodic polarisation on a steel grid in a natural marine environment in La Rochelle Harbour (left) and binocular-magnified piece of calcareous deposit after detachment of the grid (right). (**b**) Associated µ-Raman spectra. Zone 1 corresponds to the inner part of the deposit (B for brucite), and zone 2 corresponds to the outer part of the deposit (A for aragonite).

In previous work, fourteen marine bacteria able to induce CaCO_3_ precipitation were isolated from CaCO_3_-rich environments: calcareous deposit and surrounding mud and seawater [28]. In the present study, we further characterised the collected samples. The calcareous deposit formed by cathodic polarisation on steel grids immersed in seawater for 6 months is shown Figure 1a. Two strata are observed: an inner one close to the metal (zone 1) and an outer one (zone 2), which was in contact with the marine environment. The mineral composition of each stratum was determined by μ-Raman spectroscopy (Figure 1b). The spectrum of the inner stratum exhibited a vibration band around 3650 cm^−1^ corresponding to the OH symmetric vibration mode of the magnesium hydroxide (Mg(OH)_2_) named brucite. Usually, brucite also exhibits vibration bands at 280 and 450 cm^−1^ [33]. However, in our experiment, these bands were too weak compared to the fluorescence background due to organic matter and were not detected. In the outer part, aragonite, an allotropic form of CaCO_3_, was identified via its Raman vibration bands at 155, 207, and 1086 cm^−1^. The vibration band around 1085 cm^−1^ corresponds to the symmetric stretching mode of the carbonate ion and is found at a similar position in all CaCO_3_ minerals. In contrast, the weaker bands found in the 100–300 cm^−1^ region are characteristic of the crystal structure, and the peak at 207 cm^−1^ is actually characteristic of aragonite [34]. These results confirmed that the calcareous deposit formed by cathodic polarisation was composed of magnesium hydroxide (brucite) and calcium carbonate (mainly aragonite), as previously described [27,35,36]. The formation of brucite in the inner part of the deposit, i.e., closest to the polarised steel (Figure 1), is due to the high pH encountered (pH > 9.5) resulting from the polarisation and the subsequent reduction of oxygen and water [25,37]. Furthermore, aragonite was identified in zone 2, near the seawater (Figure 1), where the pH was close to that usually found for seawater, i.e., 8.2 ± 0.1. This mineral distribution corresponds to that previously reported, where the deposit consisted of an aragonite layer at the deposit/seawater interface and a magnesium-rich layer at the electrode/calcareous deposit interface [37].

**Figure 2:**
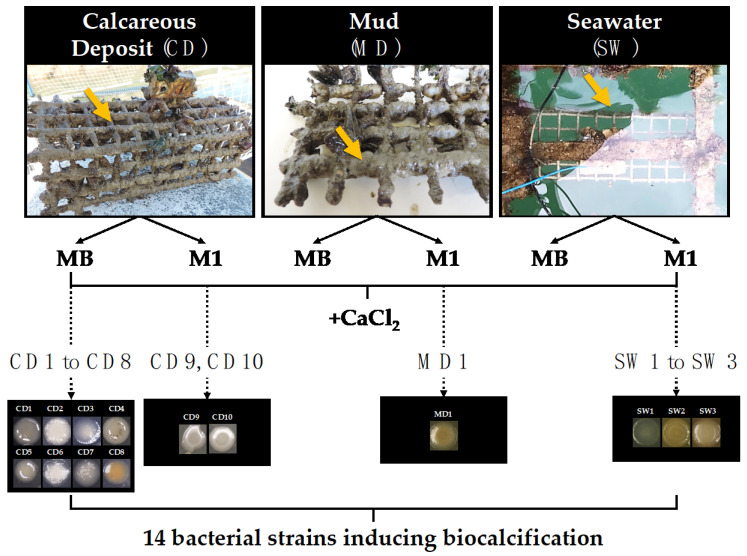
Isolation of biocalcification bacteria from the marine environment. Samples were collected on a calcareous deposit (CD) developed by cathodic polarisation on a steel grid, mud (MD), and seawater (SW) in the immediate environment of the calcareous deposit. The pictures of CD, MD, and SW are examples of samples collected in La Rochelle Harbour. Fourteen biocalcification-inducing strains were isolated and characterised (CD1 to CD10, MD1, SW1 to SW3).

The fluorescence background that appeared on the Raman spectra reflected the presence of organic matter and thus the presence of organisms (Figure 1b). This is consistent with previous works reporting that calcareous deposits formed in natural environments can be colonised by microorganisms [38,39,40,41]. However, no study had focused on the specific detection of biocalcifying bacteria within electrochemically formed calcareous deposits in marine environments until our recent preliminary study, wherein this mineral deposit formed in natural conditions was used as a potential source of biocalcifying bacteria [28]. Mud from the surface of the calcareous deposit formed on the polarised metal grid and seawater in the immediate surroundings of the deposit, as well as fragments of the deposit (zones 1 and 2, Figure 1a), was sampled as shown in the examples in Figure 2. 

After growth in liquid and solid culture media (MB or M1 enriched in CaCl_2_ to detect calcifying bacteria), bacteria from calcareous deposit, mud, and seawater samples were isolated on their respective agar media (Figure 2) [28]. Among all the bacteria that grew, only 14 colonies exhibited mineral precipitation on their surface and sometimes in the surrounding agar. These bacteria were then isolated: 10 from the calcareous deposit (CD1 to CD10 from La Rochelle Harbour), one from mud (MD from Angoulins), and three from seawater (SW from La Rochelle Harbour; SW2 and SW3 from Angoulins).

**Figure 3:**
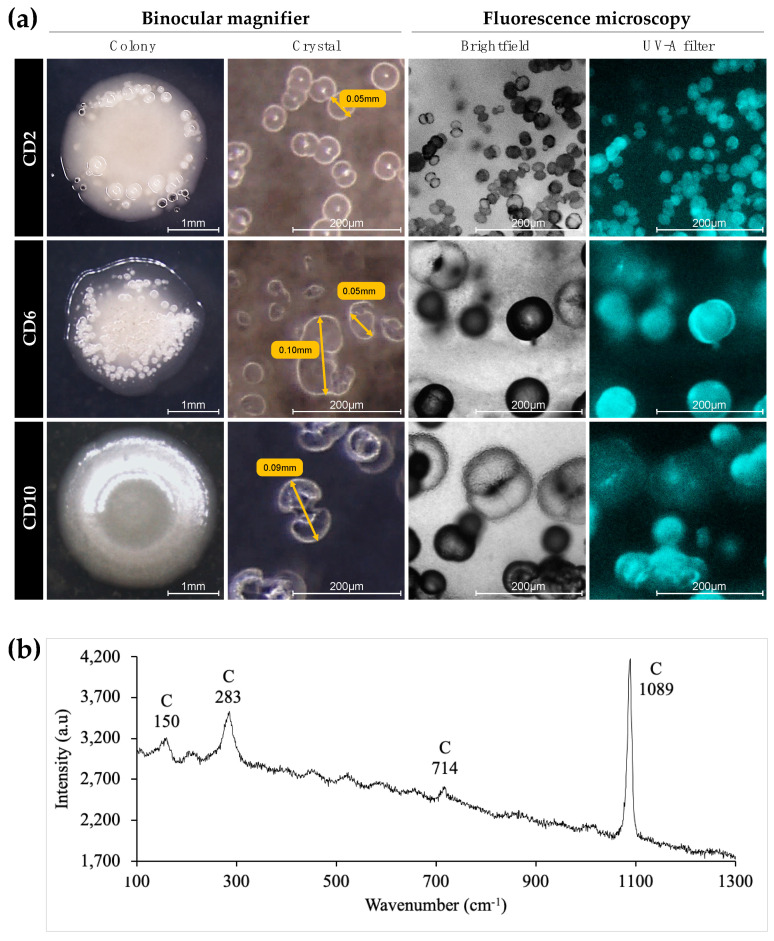
Mineral crust observed at the surface of colonies of marine bacterial strains CD2, CD6, and CD10 and associated µ-Raman spectra. (**a**) Morphology of crystals observed with binocular magnifier and fluorescence microscopy after growth on solid MB + CaCl_2_ for 3 days at 30 °C. (**b**) Example of Raman spectrum of crystals from CD10 (C for calcite).

After 3 days of growth on CaCl_2_-enriched MB plates, crystals produced by each strain were observed under a binocular magnifying glass and a fluorescence microscope, as shown in Figure 3 (CD2, CD6, and CD10 strains as examples). Crystals were clearly visible under the binocular and fluorescence microscope in brightfield, as confirmed under fluorescence, since the mineral products naturally fluoresce (Figure 3). The bacterial strains produced crystals with different shapes and sizes. CD1, CD3, CD5, CD8, CD9, CD10, MD1, SW1, SW2, and SW3 presented the most common dumbbell-shaped crystal morphology (Figure 3a for CD10). We also observed crystals forming contiguous spheres for the CD2 (Figure 3a), CD4 and CD7 strains. Crystals of the CD6 strain had an irregular form (Figure 3a). All shapes combined, the length of the crystal was between 20 µm (CD5) and 100 µm (CD6 and MD) (Figure 3a). 

Crystals of all isolated strains were analysed by µ-Raman spectroscopy [28]. Spectra obtained for all bacterial crystals corresponded to calcite, although some slight peak shifts were observed, probably due to the intense fluorescence background. The Raman spectrum of crystals from CD10 is shown as an example in Figure 3b. All Raman spectra exhibited a vibration band around 1089 cm^−1^ corresponding to the carbonate symmetric stretching mode of CO_3_^2−^. The calcite spectrum differs from that of aragonite by two vibration modes at 283 and 714 cm^−1^ instead of 207 and 705 cm^−1^ [34]. In our case, the slight shift to a higher wavenumber (1089 instead of 1085 cm^−1^) of the carbonate symmetric stretch could be due to the presence of magnesium in the calcite structure. The CaCO_3_ polymorphs resulting from biocalcification are typically calcite, aragonite, and vaterite, which are found in different shapes [4,42,43]. Calcite is the most stable form of CaCO_3_. Some researchers have suggested that the spheroidal and dumbbell shapes, as found here, reflect a microbial origin of the calcite and may serve as a bio-signature [24,44], whereas the diamond shape tends to characterise calcite formed without the influence of living organisms [45]. 

It must be noted that cathodic polarisation in seawater leads mainly to aragonite [27,35,36], while under our experimental conditions, biocalcification always led to calcite. It is generally admitted that aragonite forms in seawater because Mg^2+^ ions inhibit the growth of calcite [37]. Biocalcification takes place in the vicinity of the bacteria, in a specific environment strongly influenced by the metabolic activity of the microorganisms [3]. The nucleation-growth process of CaCO_3_ (development centre driving CaCO_3_ growth) therfore differs from that occurring in abiotic conditions and can lead to calcite instead of aragonite. 

Previous studies have shown that a wide variety of microbial genera are involved in biocalcification in various natural environments, such as soils, freshwater, oceans, salt lakes, and marine sediments [46,47,48,49,50]. To our knowledge, we are the first to have isolated a variety of marine bacteria settling calcareous deposits and their immediate surroundings, able to induce CaCO_3_ precipitation [28]. These calcareous deposits, formed on a steel grid under cathodic polarisation in marine environment, constitute a very mineral-rich and alkaline environment that may be colonised by specific consortia of bacteria able to grow and be active under these particular conditions. Ten heterotrophic marine bacteria were isolated from this very specific environment, showing their ability to resist and to grow in these extreme environmental conditions. Four other heterotrophic bacteria were also isolated from the seawater and mud in close proximity to the calcareous deposits.

### 3.2. Phylogenetic Bacterial Identification

**Figure 4:**
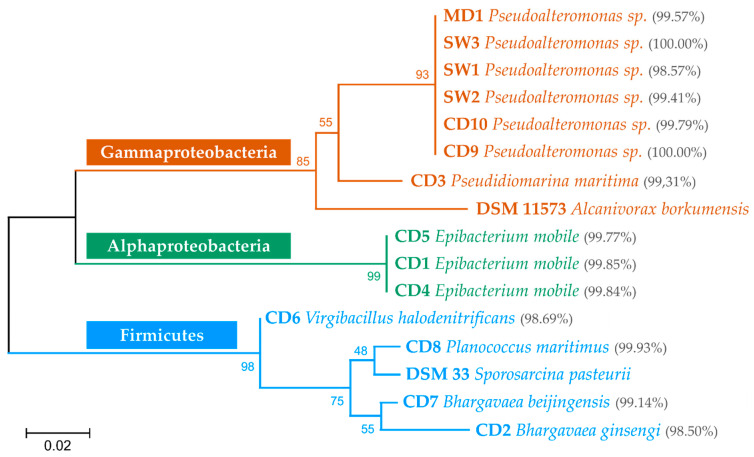
Neighbour-joining phylogenetic tree based on nearly complete 16S rRNA gene sequences of the studied strains. Bootstrap values (1000 replicates) are noted at the nodes. The percentages in brackets correspond to the highest percentages of identity between the 16S rDNA sequences of our marine strains and those of GenBank. For *Pseudoalteromonas* strains, several GenBank sequences from different species had the same percentage of identity. Therefore, only the genus was written.

The 14 biocalcifying bacterial strains were identified by sequencing the near-complete 16S rRNA gene and comparing with GenBank, SILVA ACT, and RDP II sequences. All sequences matched with at least one identified strain with more than 98.5% identity (Figure 4).

Based on their phylogenetic relationship, the isolates were divided into three groups: Gammaproteobacteria (50%), Alphaproteobacteria (21.4%), and Firmicutes (28.6%) (Figure 4). In the Gammaproteobacteria class, six isolates (MD1, SW1 to SW3, CD9 and CD10) were assigned to the *Pseudoalteromonas* genus and CD3 to the *Pseudidiomarina maritima* species. These sequences were related to *Alcanivorax borkumensis*, a marine strain selected for its ability to biocalcify and used as a reference in this study [30]. The Gammaproteobacteria group, which included 7 of the 14 bacterial strains isolated, was the largest group. The Alphaproteobacteria included three isolates (CD1, CD4, and CD5), which were all assigned with the *Epibacterium mobile* species. The Firmicutes included four isolates (CD2, CD7, CD6, and CD8) close to *Sporosarcina pasteurii*, a soil bacterium, also used as a control for its ability to biocalcify [8,9]. In this phylum, CD2 and CD7 had the closest relative belonging to *Bhargavaea* sp., and the two isolates, CD6 and CD8, were affiliated with *Virgibacillus halodenitrificans* and *Planococcus maritimus* species, respectively. We mainly identified Gram-negative bacilli (71.4%, Gammaproteobacteria and Alphaproteobacteria) but also Gram-positive bacilli (21.4%, Firmicutes) and Gram-positive cocci (7.1%, Firmicutes), microorganisms that are quite phylogenetically distant but all capable of mineralising (Table 1).

*Pseudoalteromonas* sp. was found to be the main cultured species (six strains). Possessing Gram-negative cell walls, all *Pseudoalteromonas* strains formed rod-shaped cells and possessed aerobic and chemoheterotrophic metabolism, confirming previously described the characteristics of this microorganism [51]. The *Pseudoalteromonas* genus was commonly isolated from various marine environments, such as seawater, algae, and marine invertebrates [52,53,54]. These Gammaproteobacteria are of great interest in biotechnology because of their prolific metabolite-producing ability [55] and their bioremediation potential [56]. However, few studies have shown biomineralisation by this bacterial genus. Recently, *Pseudoalteromonas lipolytica* was studied to protect steel from corrosion in seawater via the formation of a biomineralised film [57,58]. The hybrid biofilm was composed of multiple layers of calcite and extracellular polymeric substances (EPS), exhibiting high and stable barrier-protection efficiency. *Pseudoalteromonas* sp. was also shown to precipitate other minerals, such as barite (BaSO_4_), in conjunction with EPS production [54]. Herein, the biocalcifying *Pseudoalteromonas* isolates (MD1, SW1 to SW3, CD9 and CD10) were characterised by highly mucoid colonies, suggesting significant production of EPS associated with CaCO_3_.

In our work, only one strain (CD3) of the *Pseudidiomarina maritima* species, also known as *Idiomarina maritima*, was isolated for its ability to biocalcify. The CD3 strain was Gram-negative and rod-shaped and shared many phenotypic characteristics with other heterotrophic, oxidative, and marine members of the Gammaproteobacteria group. The *Pseudidiomarina* genus is widely distributed in marine and hypersaline habitats [59]. Previously, Gonzalez-Munoz et al. [60] showed that *Idiomarina* bacteria were related to biomineralisation processes by investigating their production of Mg-rich minerals (struvite (NH_4_MgPO_4_·6H_2_O) and kutnahorite (CaMg(CO_3_)_2_)) under seawater salinity conditions. *Idiomarina* sp. was also shown to precipitate barite under experimental conditions [54]. Herein, we showed, for the first time, the production of calcite by *Pseudidiomarina/Idiomarina* bacteria.

In the Alphaproteobacteria class, *Epibacterium mobile* isolates (CD1, CD4 and CD5), formerly known as *Ruegeria mobilis* [61], were Gram negative, short, rod-shaped cells that were facultative aerobic, chemoheterotrophic, catalase, and oxidase positive. The *Epibacterium mobile* species belongs to the *Roseobacter* clade, which constitutes up to 25% of the total bacterial community in coastal areas [61,62]. With this work, this species was shown, for the first time, to be involved in biocalcification. 

All Gram-positive bacilli and cocci identified in the Firmicutes phylum were facultative and anaerobic and exhibited oxidase and catalase activities. Among these strains, *Virgibacillus halodenitrificans* (formerly known as *Bacillus halodenitrificans*), *Bhargavaea beijingensis* (formerly known as *Bacillus beijingensis*) and *Bhargavaea ginsengi* (formerly known as *Bacillus ginsengi*) were all related to *Bacillus* genus. *Bacillus* bacteria are widely distributed in natural environments and have been broadly described for their ability to precipitate calcium carbonate [3,63]. Its easy cultivation and its ability to biomineralise calcite and to absorb heavy metals have made this genus one of the most studied for biomineralisation as a biotechnological tool for the construction industry and bioremediation [3,64]. Several strains affiliated with the *Virgibacillus* and *Bacillus* genera have already been isolated from brine and seawater and have been shown to form calcium carbonate minerals [29]. Concerning the *Planococcus maritimus* species, to our knowledge, so far, no work has demonstrated its capacity to biocalcify.

### 3.3. Metabolic Characterisation of the Biocalcifying Bacteria 

Ureolysis is an enzymatic pathway identified as playing a key role in the microbial biocalcification process [12,13,14]. Ureolytic bacteria produce ammonia and carbonate ions by hydrolysing urea, leading to an increase in pH and thus favouring CaCO_3_ precipitation. The presence or absence of urease activity in the 14 bacterial strains was determined by an enzymatic colorimetric assay (indole urea medium). A first analysis had shown that some strains possessed urease activity, thanks to a colorimetric test inducing a more or less intense pink coloration in the presence of urease [28]. We implemented two different protocols and increased the concentration of bacteria in the test by adding a centrifugation step in order to increase the sensitivity of this colorimetric test. In this way, we were able to demonstrate that six strains possessed urease activity: CD1, CD6, CD9, CD10, MD1, and SW2 (Table 1). The CD6 strain had the strongest urease activity. Indeed, the colour change in indole urea medium was instantaneous for this strain, regardless of the cell quantity tested. For strains CD1, CD9, CD10, MD1, and SW2, a higher concentration of cells was required to observe a slow colour change, suggesting a lower level of urease activity. Despite the use of urea-free culture media, we isolated six urease-positive strains out of 14 biocalcifying strains, distributed between *Proteobacteria* and *Firmicutes*. These results are consistent with a broad distribution of ureolysis metabolism among bacteria [50]. Urease activity creates an alkaline environment to facilitate calcium carbonate precipitation in natural settings and thus partially contributes to marine lithifications [50]. However, the six urease-positive and eight urease-negative strains produced CaCO_3_ in the absence of urea (Figure 3), suggesting that another metabolism was involved in biocalcification. The urease pathway was shown to be sufficient to induce CaCO_3_ precipitation [12,15,65], but it was also reported that in the process of biocalcification, this pathway can be combined with others, based on enzymes such as carbonic anhydrase (CA) [11,19,66]. Therefore, we explored the presence of CA activity for all isolated strains. 

Using *p*-NPA as a colour indicator during its hydrolysis by CA activity, in a previous preliminary work, we observed that all isolated marine strains would exhibit CA activity [28]. For the present study, we improved the test to make the colour change more distinct, and this new procedure confirmed the previous results (Table 1). These results were not surprising because carbonic anhydrases were reported as widespread enzymes in metabolically diverse species from Bacteria and Archaea domains, indicating that these enzymes play an extensive and fundamental role in prokaryotic biology [67,68]. Bacteria produce enzymes belonging to three distinct classes of CA, α, β, and γ, which have no significant sequence or structural identity [69]. CA are major enzymes that play a significant role in the carbon-concentrating mechanism and sequestration of CO_2_ into calcium carbonate [70,71,72]. It has been reported that this enzyme alone can accelerate the rate of CaCO_3_ formation by releasing carbonate and bicarbonate ions in large quantities [73,74]. Calcite formation and CO_2_ sequestration were successfully achieved with *Bacillus* sp. possessing enhanced activity of CA, as well as with purified extracellular CA [75]. Thus, the detection of CA activity in the 14 biocalcifying strains could explain the formation of CaCO_3_ in urease-negative and urease-positive strains without urea in the medium. 

**Figure 5:**
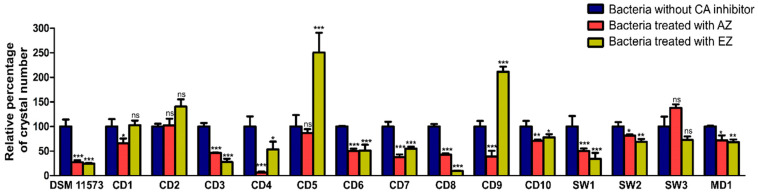
Relative crystal abundance in the biocalcification-inducing bacterial strains compared to the positive control, *Alcanivorax borkumensis* DSM 11573. Bacteria treated with 100 µmol L^−1^ of carbonic anhydrase (CA) inhibitor acetazolamide (AZ) (red) and bacteria treated with 100 µmol L^−1^ of CA inhibitor ethoxzolamide (EZ) (yellow) were compared to bacteria not treated with CA inhibitor (control, dark blue). Crystal numbers were determined per surface unit and converted to percentage. The data represent the mean values ± SD of five microscope observations for each experiment. The 100% values on the *Y*-axis correspond to the crystal number of the controls. Two-way ANOVA and Bonferroni post hoc test were used for statistical analysis. Significant differences are indicated by * (*p* < 0.05), ** (*p* < 0.01), or *** (*p* < 0.001) when AZ or EZ conditions are compared to the control. ns: not significant.

To validate the CA activity and its role in biocalcification, growth experiments with two sulphonamide CA-inhibitors (acetazolamide and ethoxzolamide) were performed for the 14 biocalcifying strains (Figure 5).

Both inhibitors are specific to CA inactivation [30,69]. The addition of either acetazolamide (a membrane-impermeable compound) or ethoxzolamide (a membrane-permeable molecule) to the 14 strains inoculated on agar medium resulted in a change in the amount of CaCO_3_ crystals for the majority of strains (Figure 5). The nine strains, CD3, CD4, CD6, CD7, CD8, CD10, SW1, SW2, and MD1, showed a significant decrease in crystal production in the presence of both inhibitors, as observed for the positive control, *Alcanivorax borkumensis* DSM 11573. CD9 also showed a significant reduction in crystal abundance but in presence of acetazolamide inhibitor only. These results support the direct involvement of CA activity in CaCO_3_ formation for these bacteria. Conversely, for CD9, an increase in the crystal number was observed in presence of ethoxzolamide (Figure 5). CD8 also showed a metabolic change in colony development in the presence of ethoxzolamide, as this strain was no longer had an orange pigmentation. Crystals could also appear larger than in the control condition, a phenomenon mainly observed with the CD10 strain. As CA is fundamental to many prokaryotic biological processes, its inhibition could influence the growth of microorganisms [76,77,78]. Moreover, we observed that the inhibition of crystal production by one or both CA inhibitors was never complete (Figure 5). Two hypotheses could explain this phenomenon. First, the inhibitors would not have a strong affinity for CA of our bacterial strains. In this case, the enzyme would be minimally inhibited and would therefore continue to induce CaCO_3_ formation. This case is quite possible, as it has been shown that affinities for sulphonamide inhibitors are highly variable, depending on CA [79]. The second possibility is that other metabolisms would be involved in biocalcification and could act synergistically with CA activity or could take over. Our data show that the six urease and CA positive strains, CD1, CD6, CD9, CD10, MD1, and SW2, could couple the action of both enzymes. The synergy of these two metabolic pathways would allow for a better efficiency of biocalcification. Indeed, CaCO_3_ precipitation induced by CA activity could be enhanced by the alkaline conditions provided by urease activity [45,66]. Moreover, for CD2, CD5, and SW3, the urease-negative strains, we did not demonstrate a direct action of CA in CaCO_3_ crystal formation, as the enzyme inhibitors used did not significantly reduce the number of crystals. This suggests that either the CA of these strains has no affinity for these inhibitors, or that CA and urease pathways may not be the only mechanisms involved in biocalcification. Further experiments will have to be carried out to highlight the metabolic pathways involved in this biocalcification.

### 3.4. Analysis of Biocalcification Capacity

**Figure 6:**
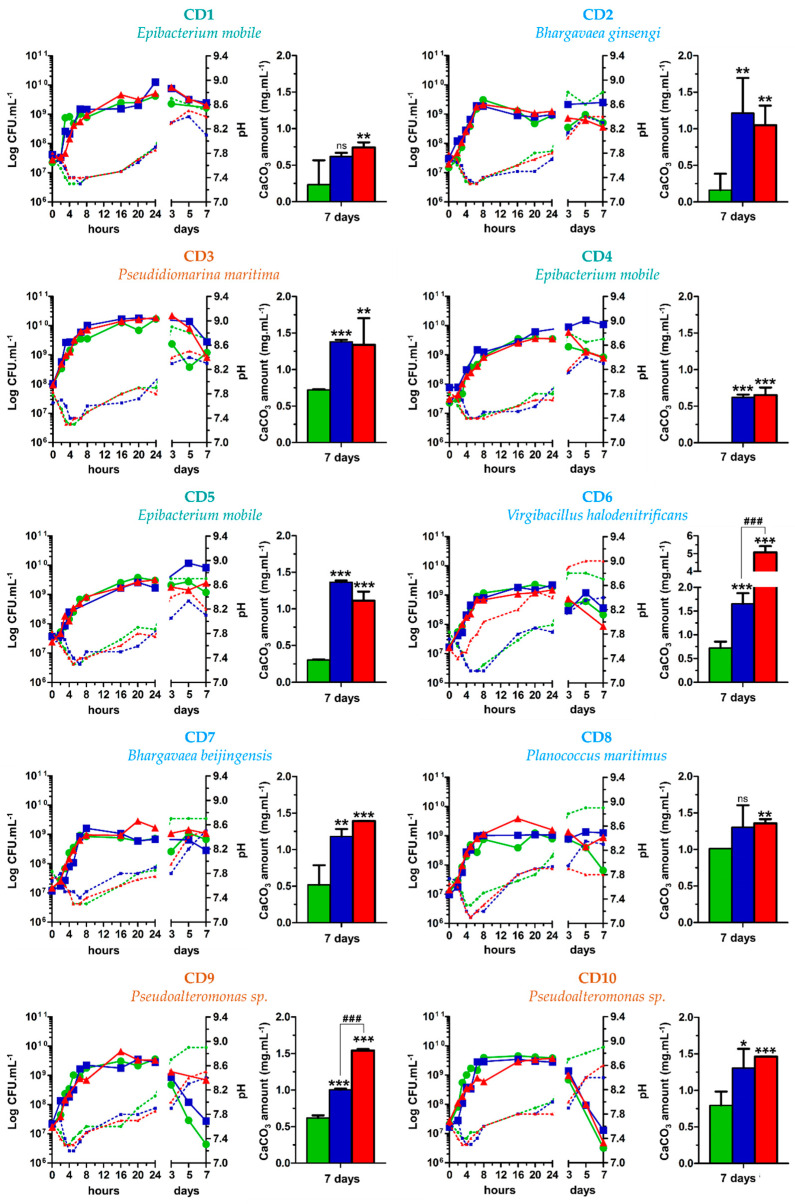

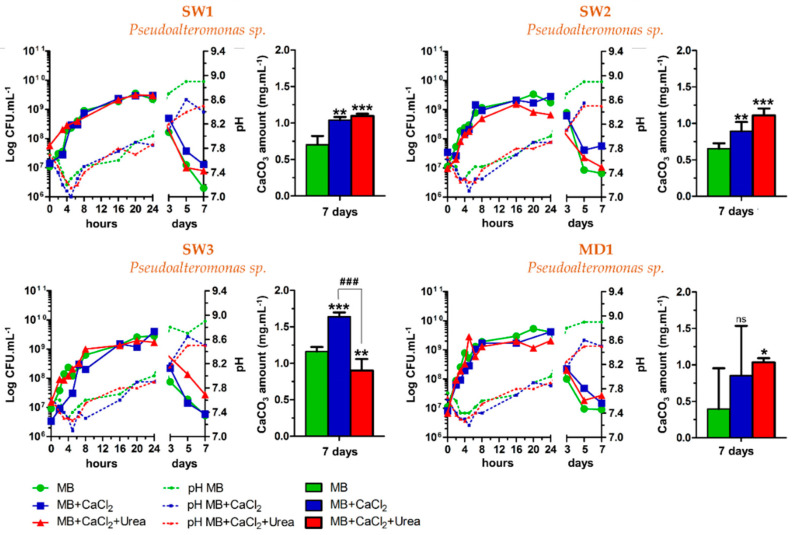
Bacterial growth, pH monitoring, and capability of inducing CaCO_3_ precipitation of the 14 isolated marine bacteria and the control, *Sporosarcina pasteurii* DSM 33. Bacterial growth was monitored in MB (green), MB + CaCl_2_ (blue), and MB + CaCl_2_ + urea (red) for 7 days at 30 °C. Two-way ANOVA and Bonferroni post hoc test were used for statistical analysis. Significant differences are indicated by * (*p* < 0.05), ** (*p* < 0.01), or *** (*p* < 0.001) when MB condition (green) is compared to MB + CaCl_2_ (blue) or to MB + CaCl_2_ + urea (red), and not significant differences are indicated by ns. Significant differences are indicated by ### (*p* < 0.001) when MB + CaCl_2_ condition (blue) is compared to MB + CaCl_2_ + urea (red).

In order to evaluate the performance of the different isolated strains regarding their CaCO_3_ production, bacterial growth with continuous monitoring of pH evolution and quantification of CaCO_3_ formed at 7 days of incubation (to have enough CaCO_3_) was performed in the following liquid culture media: MB, MB + CaCl_2_, and MB + CaCl_2_ + urea (Figure 6).

Obviously, no changes in pH or crystal formation were detected in the control experiments without bacteria. In the liquid media, all strains induced calcite precipitation (except the CD4 strain in MB) and grew rapidly to reach maximum growth between 8 and 16 h of incubation, regardless of culture conditions (Figure 6). Since no CaCO_3_ precipitation was observed in the control experiments of non-inoculated media, the importance of microbial activity in biocalcification was demonstrated. After 3 days, most strains showed a decline phase, indicating that cell viability was not maintained over time, and thus, CaCO_3_ production was potentially lower over longer periods without medium renewal. Furthermore, the enrichment of the culture media with CaCl_2_ and urea had no impact on bacterial growth, even over longer periods. Indeed, the growth curves obtained for the three culture conditions were similar for each strain. These results showed that the nucleation of CaCO_3_ crystals induced by the bacteria did not affect their active growth phase. It has been reported that bacteria also play an important role in mineral precipitation by providing abundant reactive sites in the EPS, cell walls, and external sheaths of bacterial cells that bind dissolved mineral-forming elements [4,24]. Nucleation sites are present on bacterial cell walls since ion exchange can occur through the cell membrane [63]. The EPS matrix secreted by bacteria also represents an extension for the microbial cell and functions as a chelator for cations. Thus, this matrix can be the template for crystal nucleation [80,81,82]. Under alkaline conditions, the amino acids associated with the EPS are deprotonated and can adsorb Ca^2+^ and Mg^2+^ ions [4]. Crystals may then nucleate and grow from cation-enriched solutions on the outer surface of individual cells [48,60]. In our work, since CaCO_3_ precipitation had no impact on bacterial growth and no mineral shells surrounding the bacteria were observed (data not shown), it would therefore appear that the nucleation sites are not on the cell walls but rather correspond to the diffuse EPS outside the cells.

pH was also monitored for the three culture conditions for 7 days (Figure 6). For all isolated strains, a slight acidification of the medium was measured during the first hours of the exponential growth phase. During the exponential phase of growth, the metabolic activity of bacteria is optimal. It can be assumed that metabolic pathways induced an acidification during this phase. Castro-Alonso et al. suggested that an acidification step of culture medium occurs during biocalcification [3]. Indeed, under unfavourable conditions caused by high Ca^2+^ concentration, the cell survives by allowing the entry and accumulation of calcium ions, resulting in an excessive expulsion of protons. Subsequently, the cells actively export calcium and compensate for the loss of protons. A low concentration of protons and a high concentration of Ca^2+^ in the microenvironment is required for secretion of carbonate ions, while supersaturation of carbonate induces precipitation of calcium carbonate on the surface of the cell [3]. pH tends to increase under the action of bacterial metabolism with urease, CA, and/or other enzymes. Indeed, the bacteria responsible for the biocalcification phenomenon are known to induce alkalisation of their environment, thus promoting CaCO_3_ precipitation [3,5]. After 24 h of incubation, the pH measured for all strains reached 7.8 to 8. This result is consistent with our observations of visible crystals on the surface of colonies on agar medium after 24 h of incubation. Regarding the pH evolution on the seventh day of the bioassay, a direct relationship was observed between higher final pH levels and higher concentrations of total CaCO_3_ precipitated in the two calcium-enriched conditions. Thus, in the presence of urea, the urease-positive CD6 strain exhibited pH values (pH 9) and CaCO_3_ production (5.1 mg mL^−1^) significantly higher than those of the other strains. These results, together with the urease activity assay result (Table 1), imply that the mass of CaCO_3_ precipitated was directly related to urease activity for the urease-positive strain *Virgibacillus halodenitrificans* CD6, with higher urease activity resulting in greater CaCO_3_ production. This result coincides with the observations of Hammes et al. [83], who reported a diversity of urease genes in the genomes of ureolytic bacteria and proposed that their high affinities and specific rates were the basis for rapid crystal formation. 

The composition of the culture medium also influenced the bacterial production of CaCO_3_ (Figure 6). The MB medium used without enrichment contains a CaCl_2_ concentration equivalent to that of seawater. This CaCl_2_ concentration is sufficient to allow the bacteria to produce a concentration of CaCO_3_ quantifiable by thermogravimetric analysis, except for the strain CD4 (Figure 6). For all strains, CaCl_2_ enrichment of the MB culture medium led to significantly higher CaCO_3_ production compared to unenriched MB. Indeed, an increase in dissolved calcium concentration in the medium is known to increase bacterial CaCO_3_ production [84]. Nevertheless, for the same dissolved calcium concentration, isolated biocalcifying strains did not produce the same amount of CaCO_3_ (Figure 6). CD1 (urease-positive, CA-positive) and CD4 (urease-negative, CA-positive) produced 0.62 mg mL^−1^ CaCO_3_, while SW3 (urease-negative, CA-positive) produced 1.65 mg mL^−1^. Their differing ability to biocalcify in this environment may be due to their different metabolic capacities. The absence of urea in the medium suggests that CA and/or a metabolic pathway other than urease are effective in inducing CaCO_3_ precipitation. Urea enrichment (MB + CaCl_2_ + urea) was used to assess the biocalcification capacity in the presence of urea for both urease-positive and urease-negative strains (Figure 6). Based on our data, urea enrichment had no impact on CaCO_3_ production for urease-negative strains, except for SW3. Indeed, CaCO_3_ production by the SW3 strain was significantly lower in the presence of urea. Among the six urease-positive strains (CD1, CD6, CD9, CD10, MD1, and SW2), CaCO_3_ production in the presence of urea was significantly higher for only two strains—CD6 (5.1 mg mL^−1^) and CD9 (1.5 mg mL^−1^) (Figure 6)—confirming the differences in urease activity and thus CaCO_3_ production among the different bacteria. CD6 was the only strain to have similar CaCO_3_ production to that produced by the urease-positive control, *Sporosarcina pasteurii* DSM33 (5.3 mg mL^−1^). *Sporosarcina pasteurii* is an efficient ureolytic strain extensively studied in CaCO_3_ production mechanisms and in various biomineralisation applications [3,8,9,12,15,85]. Thus, in our work, the *Virgibacillus halodenitrificans* CD6 urease-positive strain was the most efficient marine strain to induce calcium carbonate precipitation, with an ability equivalent to that of the related bacterium *Sporosarcina pasteurii*, the well-known biocalcifying bacterium described in the literature. Recently, Arias et al. [46] also described halotolerant ureolytic bacteria isolated from Laguna Salada with slightly higher (≥6 mg mL^−1^) biocalcification capabilities than those of CD6. These isolates were identified as *Bacillus* (related to *Sporosarcina*), *Salinivibrio*, and *Halomonas* species, already known to be biocalcifying [46]. Concerning the urease-negative strains, CaCO_3_ production with CA activity was overall lower than that of CD6 in the presence of urea, while the *Pseudoalteromonas* SW3 strain was, nevertheless, as efficient as CD6 under conditions without urea (1.7 mg mL^−1^) (Figure 6). This result confirms the observations of Dhami et al. [86] that the urease pathway is faster and more efficient than the CA pathway in terms of extracellular enzyme production and calcium carbonate precipitation. Although the CA pathway was demonstrated herein to be less efficient in CaCO_3_ production overall, this metabolism has a substantial advantage over the urease pathway, namely the absence of ammonia production, a toxic molecule that could limit the use of the ureolytic pathway [87].

Finally, the metabolic properties of these biocalcifying marine bacteria lead to the formation of precipitated CaCO_3_ may act as a bio-cement [86]. Indeed, CaCO_3_ could be used as a natural cement, binding sediments, sand, shells, etc., giving rise to a solid agglomerate. In recent years, cementing bacteria have been widely studied for applications in multiple engineering fields, including geotechnical, construction, and environmental engineering, where they are used as a means of bio-cementation for the strengthening and consolidation of soils and sand or for the protection and restoration of stone and concrete structures [3,12,86,88]. Thus, the process of bio-cement formation by biocalcifying marine bacteria offers an exciting and innovative potential that could be synergistically combined with the electrochemical process of calcareous deposit formation [27]. Potential applications include reinforcement of natural and artificial marine structures and prevention of coastal erosion.

## 4. Conclusions

In conclusion, we showed that calcareous deposits formed on a steel grid under cathodic polarisation in marine environment harbour bacteria belonging to different genera that are able to induce CaCO_3_ precipitation. The ability to grow in an alkaline and saline environment is crucial for calcium-carbonate-precipitating bacteria to be used in such environments. We showed that the composition of the medium, in particular the concentration of CaCl_2_, influenced the amount of CaCO_3_ produced by the 14 bacterial strains. Moreover, urease-positive bacteria showed higher CaCO_3_ production than urease-negative bacteria, as observed for the more efficient strain, *Virgibacillus halodenitrificans* CD6, in the presence of urea. Carbonic anhydrase—and potentially other enzymes—would be also involved in biocalcification and could act in synergy with urease metabolism, depending on the strain. Thus, these marine bacteria could be promising candidates for eco-friendly technological applications, such as the formation of bio-cements in synergy with the electrochemical process of calcareous deposit formation, in order to reinforce marine structures and to delay coastal erosion. 

## Figures and Tables

**Table 1:** Characterisation of isolated biocalcifying bacterial strains. Marine bacteria grew for 3 days on solid MB at 30 °C. Identification, morphotype, urease, and carbonic anhydrase production are presented below.

Identification	Morphotype	Urease	Carbonic Anhydrase
CD1 *Epibacterium mobile*	Gram-negative bacilli	+	+
CD2 *Bhargavaea ginsengi*	Gram-positive bacilli	-	+
CD3 *Pseudidiomarina maritima*	Gram-negative bacilli	-	+
CD4 *Epibacterium mobile*	Gram-negative bacilli	-	+
CD5 *Epibacterium mobile*	Gram-negative bacilli	-	+
CD6 *Virgibacillus halodenitrificans*	Gram-positive bacilli	+	+
CD7 *Bhargavaea beijingensis*	Gram-positive bacilli	-	+
CD8 *Planococcus maritimus*	Gram-positive bacilli	-	+
CD9 *Pseudoalteromonas* sp.	Gram-negative bacilli	+	+
CD10 *Pseudoalteromonas* sp.	Gram-negative bacilli	+	+
MD1 *Pseudoalteromonas* sp.	Gram-negative bacilli	+	+
SW1 *Pseudoalteromonas* sp.	Gram-negative bacilli	-	+
SW2 *Pseudoalteromonas* sp.	Gram-negative bacilli	+	+
SW3 *Pseudoalteromonas* sp.	Gram-negative bacilli	-	+

## Data Availability

Not applicable.
